# Editorial: Micro/nano devices and technologies for neural science and medical applications

**DOI:** 10.3389/fbioe.2024.1545853

**Published:** 2025-01-06

**Authors:** Juntao Liu, Zhugen Yang, Yang Wang, Li Wang, Ziyue Li

**Affiliations:** ^1^ Aerospace Information Research Institute, Chinese Academy of Sciences, Beijing, China; ^2^ Centre for Water, Environment and Development, Cranfield University, Cranfield, Oxfordshire, United Kingdom; ^3^ School of Biological Science and Medical Engineering, Beihang University, Beijing, China; ^4^ Faculty of Mechanical Engineering, Qilu University of Technology, Ji’nan, China; ^5^ University of Connecticut Health Center, University of Connecticut, Storrs, CT, United States

**Keywords:** micro/nano devices, neural science, brain computer interface, nanotechnology, life science

Research on micro/nano devices and technologies represents a significant Frontier at the intersection of information science and life sciences, holding substantial strategic importance and promising application prospects in the fields of neural science and medical applications (Liu et al., 2020). With the rapid advancement of micro/nano processing technology, innovative intelligent, miniaturized, and integrated devices are emerging. These devices offer distinct advantages in detection and regulation. Notably, integrating micro/nano devices with neural science and clinical medicine can address scientific frontiers while fostering new research hotspots.

Epilepsy is a major neurological disorder that affects over sixty million individuals worldwide, severely impacting their health and quality of life (Bernhardt et al., 2019). To elucidate the mechanisms underlying epilepsy as well as its treatment options, it is essential to investigate changes in neural activity within relevant neural circuits. Implantable microelectrode arrays—capable of high-quality signal recording and neural information decoding—exhibit considerable potential for application in brain-computer interfaces (Wang et al., 2024). Han et al. designed and fabricated an implantable microelectrode array specifically for electrophysiological signal detection and analysis within the striatal region of the basal ganglia in epileptic rats. The analysis of electrophysiological data from the striatum during episodes of epilepsy provides valuable insights into the dynamic processes governing striatal neural activity during both initial onset phases as well as latent periods associated with temporal lobe epilepsy. This understanding contributes to unraveling the neural mechanisms involved in epilepsy while promoting advancements in related therapeutic approaches for this condition.

Pain is an emotional and unpleasant sensory experience that can lead to significant physiological and psychological effects across various aspects of life and work. Recent advancements in nanotechnology have paved the way for innovative pain relief strategies utilizing diverse nanomaterials and targeted surface modifications (Shi et al., 2023). Zhu et al. summarized the status of the research and global trends of nanotechnology in relation to pain management. Neuromodulation represents another therapeutic approach for alleviating pain by modulating abnormal nerve activity through light, sound, or electrical stimulation. Nanomaterials such as carbon nanotubes, graphene, piezoelectric ceramics, and magnetic metals have garnered extensive research interest for use in implantable or wearable devices designed for neuromodulation. In conclusion, emerging novel drugs and technologies present a wide array of options for effective pain management.

The detection of biomarkers holds significant importance for disease diagnosis and treatment as well as life science research (Sun et al., 2022). Biosensors are specialized sensors capable of detecting a broad spectrum of biological and chemical substances. The integration of nanotechnology into the field of biosensors has enhanced their sensitivity along with other critical properties, leading to the development of innovative biosensing solutions. Focusing on E2 specifically, Wang et al. reviewed the extensive applications of new functional nanomaterials within various biosensors while discussing whether modified bioreceptors or energy exchange interfaces enable these functional nanomaterials to enhance sensing performance through improved signal conversion rates, signal amplification capabilities, and biocompatibility.

Nanoenzymes represent a novel class of nanomaterials exhibiting enzyme-like activity that has attracted considerable attention within the biomedical field in recent years. Compared to traditional biological enzymes, nanoenzymes demonstrate superior stability alongside greater heat resistance and broader pH adaptability ranges; these characteristics underscore their substantial potential in various applications (Christodoulou, et al., 2023). Chen et al. classified metal-organic framework (MOF)-based nanozymes, analyzed their application in stroke treatment and highlighted the challenges in developing efficient antioxidant nanozyme systems for stroke treatment.

Micro-nano technology is currently at the forefront of technological advancement, significantly enhancing detection and regulation capabilities. We can leverage micro-nano technology to develop new devices and equipment tailored to specific needs. To address the demand for deep brain detection and regulation, it is essential to create miniaturized, high-throughput implantable brain-computer interfaces (Yang et al., 2024). In response to point-of-care testing (POCT) requirements, we must focus on developing biosensors and devices that offer rapid detection times, high sensitivity, and user-friendly operation. Utilizing micro-nano technology allows for the design and development of innovative miniaturized sensor devices capable of achieving previously unattainable levels of detection. For instance, the nervous system transmits information through two fundamental modes: electrical signals from nerve cells and chemical transmitters (dual-mode). The dual-mode neural microelectrode array can mitigate information loss associated with single-mode recording. This advancement holds significant scientific importance and application value in researching neural diseases and advancing our understanding of brain science. We believe that through ongoing development and innovation, these technologies will play a pivotal role in driving progress within neuroscience and clinical medicine.
